# Nickel Oxide Decorated Halloysite Nanotubes as Sulfur Host Materials for Lithium–Sulfur Batteries

**DOI:** 10.1002/gch2.202300005

**Published:** 2023-05-13

**Authors:** Meltem Karaismailoglu Elibol, Lihong Jiang, Dongjiu Xie, Sijia Cao, Xuefeng Pan, Eneli Härk, Yan Lu

**Affiliations:** ^1^ Department for Electrochemical Energy Storage Helmholtz‐Zentrum Berlin für Materialien und Energie Hahn‐Meitner Platz 1 14109 Berlin Germany; ^2^ Department for Energy Science and Technology Turkish‐German University Şahinkaya Cad. 106 İstanbul 34820 Turkey; ^3^ Key Laboratory of Textile Science & Technology College of Textiles Donghua University North Renmin Road 2999 Shanghai 201620 P. R. China; ^4^ Institute of Chemistry University of Potsdam Karl‐Liebknecht‐Straße 24‐25 14476 Potsdam Germany

**Keywords:** cycling stability, halloysites, lithium–sulfur batteries, nickel oxide

## Abstract

Lithium–sulfur batteries with high energy density still confront many challenges, such as polysulfide dissolution, the large volume change of sulfur, and fast capacity fading in long‐term cycling. Herein, a naturally abundant clay material, halloysite, is introduced as a sulfur host material in the cathode of Li–S batteries. Nickel oxide nanoparticles are embedded into the halloysite nanotubes (NiO@Halloysite) by hydrothermal and calcination treatment to improve the affinity of halloysite nanotubes to polysulfides. The NiO@Halloysite composite loaded with sulfur (S/NiO@Halloysite) is employed as the cathode of Li–S batteries, which combines the physical confinements of tubular halloysite particles and good chemical adsorption ability of NiO. The S/NiO@Halloysite electrode exhibits a high discharge capacity of 1205.47 mAh g^−1^ at 0.1 C. In addition, it demonstrates enhanced cycling stability, retaining ≈60% of initial capacity after 450 cycles at 0.5 C. The synthesized NiO@Halloysite can provide a promising prospect and valuable insight into applying natural clay materials in Li–S batteries.

## Graphical abstract

Halloysite, a kind of natural clay material, is used as a template to synthesize nickel oxide@halloysite composites, which are utilized as sulfur host materials in the cathode of lithium–sulfur batteries. The constructed cathodes exhibit a high specific discharge capacity of 1205.47 mAh g^−1^ at 0.1 C, good reversibility, and long‐term cycling performance.

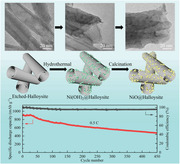

## 1 Introduction

The concerns about increasing consumption of fossil fuels, global warming, and other environmental issues have stimulated the development of sustainable, environmentally friendly, and safe energy storage technologies.^[^ ^1^ ^]^ Among all the sustainable energy systems, rechargeable batteries are reliable systems that can store and release energy electrochemically in an efficient way.^[^ ^2^ ^]^ The present studies show that the use of batteries is increasing with the demand for energy storage.^[^ ^3^ ^]^ Significantly, the development of batteries with high energy densities is required to meet the demand for electric vehicles.^[^ ^4^ ^]^ Although lithium‐ion batteries dominate the market of portable electronic devices these years, the limited energy density and safety issue of cathodes impede their further development and applications.^[^ ^5^ ^]^ Therefore, other energy storage systems and technologies with enhanced energy density, low cost, as well as high safety have been intensively explored. Lithium–sulfur (Li–S) batteries have attracted increasing attention because of the high theoretical specific energy density of 2600 Wh kg^−1^.^[^ ^6^ ^]^ However, due to the electrically insulating sulfur in the cathode of Li–S batteries, the utilization of appropriate host materials is still essential to enhance the electronic conductivity and electrochemical performance of sulfur host cathode.^[^ ^7^ ^]^ Moreover, various intermediate products of lithium polysulfides (LiPSs) are generated during the electrochemical reaction process, among which the high‐order polysulfides are easily soluble in the ether‐based electrolyte, as well as diffuse to the lithium anode to be reduced to Li_2_S_2_ and Li_2_S species.^[^ ^8^ ^]^ The shuttle effect leads to the reduction of sulfur's utilization rate, inferior Coulombic efficiency and fast capacity fading.^[^ ^7^, ^9^ ^]^

In response to these challenges, various strategies have been used to develop the host materials and suppress the shuttle effect. Some research studies have focused on impregnating sulfur particles in porous carbon materials, impeding the diffusion of polysulfides from the cathode side by physical confinements to improve electrochemical reversibility.^[^ ^10^ ^]^ Even though physical confinements enhance electrochemical performance during initial charge/discharge cycles, these improvements typically decline rapidly in subsequent cycles due to the relatively weak interactions between low‐polarity carbon and high‐polarity LiPSs^[^ ^11^ ^]^ and the inability of physical confinements to tackle the LiPSs shuttle effect.^[^ ^12^ ^]^ Therefore, using metal oxide additives in the cathode matrix and combining with chemical confinement is another effective strategy to inhibit the shuttle effect.^[^ ^13^ ^]^

Due to the low cost, abundant resources and environmental friendliness,^[^ ^14^ ^]^ halloysite mineral has been applied in many fields, such as the pharmaceutical and ceramic industry,^[^ ^15^ ^]^ tissue engineering,^[^ ^16^ ^]^ building engineering,^[^ ^17^ ^]^ catalysis,^[^ ^18^ ^]^ and cosmetics.^[^ ^19^ ^]^ Generally, the halloysite nanotubes (HNT) are 800 ± 300 nm in length and 15 ± 5 nm in outer diameter, with the outer lumen composed of silica and inner lumen containing alumina. The mesoporous silica in hollow HNT tends to adsorb polysulfide anions, which prevents their release to the electrolyte.^[^ ^20^ ^]^ Hence, using halloysite with mesoporous silica in the cathode of Li–S batteries could suppress the random transport of LiPSs to some extent and provide a high Coulombic efficiency.^[^ ^21^ ^]^ However, raw halloysite nanotubes are electrically insulating and their immobilization for LiPSs can be improved for a better battery performance.^[^ ^22^ ^]^ Hence, efforts have been made to improve its electrical conductivity and electrochemical activity by coating the materials with carbon or conductive polymers. For example, halloysite nanotubes were coated with polydopamine which is a carbon precursor, and tested as the sulfur host material (C@S/HNT) in a Li–S battery. The results indicate that the C@S/HNT cathode had a discharge capacity of 922.7 mAh g^−1^ at 0.1C, and the capacity was preserved ≈82% after charge and discharge for 500 cycles at 1 C.^[^ ^21b^ ^]^ Although the cycling properties are enhanced owing to the efficient trapping ability of highly conductive HNT for polysulfides in the charge and discharge process, the specific discharge capacity still needs to improve. Another research used reduced graphene oxide (rGO) to enhance the electrical conductivity of halloysite particles, revealing that rGO/HNTs/S composite cathode has an improved discharge capacity of 1134 mAh g^−1^ at 0.1C.^[^ ^23^ ^]^ However, the specific discharge capacity only retained 67.1% after cycling for 50 cycles at 0.1 C due to the limited confinements of rGO and HNTs to LiPSs. Therefore, it is still a challenge to explore effective strategies for halloysites modification to simultaneously enhance the discharge capacity and cycling performance of halloysite‐based cathode in Li–S batteries.

Nickel oxide has been extensively studied and applied in sensors,^[^ ^24^ ^]^ solar cells,^[^ ^25^ ^]^ and photoelectrolysis devices^[^ ^26^ ^]^ because of its electrical and optical properties. In addition, NiO was investigated as a sulfur host material in cathode of Li–S batteries to improve the electrochemical properties of the cathode, and it has been proved that the addition of nickel oxide into the sulfur structure led to an increase in the specific capacity.^[^ ^27^ ^]^ Its better electrochemical performance can be attributed to the increasing number of electroactive sites^[^ ^28^ ^]^ and good adsorption ability toward LiPSs.^[^ ^29^ ^]^ In this study, inspired by the adsorption ability of halloysite nanotubes and excellent electrochemical performance of NiO, NiO@Halloysite nanocomposite has been prepared via hydrothermal method and calcination treatment at the temperature of 550°C and subsequently used as sulfur host material (S/NiO@Halloysite) in Li–S batteries. For a better understanding of the effect of NiO particles, the electrochemical properties of S/NiO@Halloysite cathode were compared with the reference sample of halloysite without NiO. The cyclic voltammetry (CV), galvanostatic charge/discharge (GCD) and electrochemical impedance spectroscopy (EIS) measurements were conducted to investigate the cyclic stability, specific capacity, rate capability and identify the features that control electrochemical reactions. The synthesis route of NiO@Halloysite can provide a simple and scalable method for further utilization and modification of halloysites, which proposes an effective strategy for applying nanoclay materials in energy storage devices.

## 2 Results and Discussion

**Figure 1:**
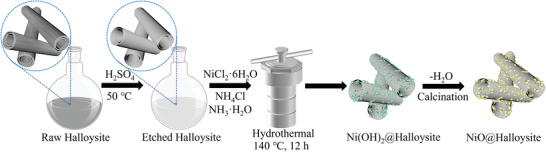
The schematic diagram for the synthesis process of NiO@Halloysite composites.

**Figure 2:**
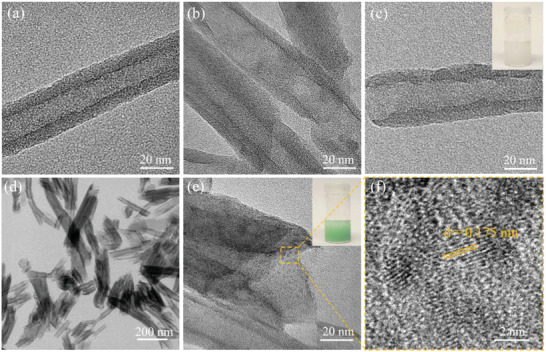
TEM images of a) raw halloysite, b) etched‐halloysite, c) etched‐halloysite after calcination at 550°C, d) *β*‐Ni(OH)_2_@halloysite composite, and e) HRTEM image of f) *β*‐Ni(OH)_2_@halloysite composite. The insets in (c) and (e) are the photographs of Etched‐Halloysite‐550 and *β*‐Ni(OH)_2_@halloysite dispersed in ethanol, respectively.

**Figure** **1** shows the synthesis procedure of NiO@Halloysite composites. First, the halloysite was etched by sulfuric acid (H_2_SO_4_) at 50°C. Then, *β*‐Ni(OH)_2_ nanoparticles were deposited on the etched halloysitenanotubes by the hydrothermal method in an ammonia solution using NiCl_2_ as a nickel precursor. The tubular structure of raw halloysite was proved by the transmission electron microscopy (TEM) image in **Figure** **2**a and Figure S1a (Supporting Information). Raw halloysite was treated with H_2_SO_4_ for the removal of alumina from its inner lumen.^[^ ^30^ ^]^ In Figure 2b and Figure S1b (Supporting Information), the tubular morphology of the etched halloysite waspreserved, and an increase in inner diameter and coarse inner wall in the halloysite was observed after the dealumination process. In addition, the etched‐halloysite was calcinated at 550°C and the product was assigned as Etched‐Halloysite‐550. Figure 2c and Figure S1c (Supporting Information) show that the tubular structure of the etched‐halloysite was preserved after calcination. Figure S2 (Supporting Information) exhibits the N_2_ adsorption–desorption curves of the raw halloysite and etched‐halloysite. The specific surface area of the halloysites was increased from 92.8 to 126.1 m^2^ g^−1^ after acid treatment. It is because the acid etches the alumina in the inner lumen of halloysite nanotubes,^[^ ^31^ ^]^ which can be seen from the coarse inner wall in Figure 2b. After hydrothermal treatment, *β*‐Ni(OH)_2_ particles were successfully synthesized on the surface of halloysite tubes, as proved in X‐ray diffraction (XRD) patterns in Figure S3 (Supporting Information).^[^ ^32^ ^]^ The *β*‐Ni(OH)_2_@halloysite's structures still appear tubular, shown in Figure S4 (Supporting Information). TEM images in Figure 2d,e confirmed that the fine *β*‐Ni(OH)_2_ nanoparticles are successfully deposited on the etched‐halloysite nanotubes. Moreover, the as‐synthesized *β*‐Ni(OH)_2_@halloysite solution shows bright green color (inset of Figure 2e), while the color of the Etched‐Halloysite‐550 is milky white (inset of Figure 2c), which further illustrates the deposition of *β*‐Ni(OH)_2_ on the halloysite. Figure 2f shows the high‐resolution transmission electron microscopy (HRTEM) image of the *β*‐Ni(OH)_2_@halloysite composite, which proves the fringe *d*‐spacing (0.175 nm) is in good agreement with the (102) atomic plane spacing of *β*‐Ni(OH)_2_ crystal phase.^[^ ^33^ ^]^ Considering the XRD patterns of the untreated halloysite, etched halloysite, and Etched‐Halloysite‐550 which are shown in Figure S3 (Supporting Information), the reflections at 2*θ* values of 11.86° and 20.14° are assigned to the presence of the dehydrated halloysite phase.^[^ ^34^ ^]^ In addition to the halloysite crystal phase, the peak at 26.64° is corresponding to SiO_2_.^[^ ^35^ ^]^ The XRD pattern of the *β*‐Ni(OH)_2_@halloysite (Figure S3, Supporting Information) demonstrates both the characteristic peaks from halloysite and *β*‐Ni(OH)_2_, confirming *β*‐Ni(OH)_2_ particles were successfully deposited on the halloysite after hydrothermal treatment. It is noted that the peak of SiO_2_ disappeared in *β*‐Ni(OH)_2_@halloysite, which could be attributed to the dissolution of the Si—O—Si network in an alkaline solution during the long‐time hydrothermal treatment. This is because SiO_2_ is soluble in alkaline solution due to the formation of silicate ions and Si(OH)_4_, and the solubility will increase with the increase of pH values (the pH value of the reaction solution in this work is ≈9.6).^[^ ^36^ ^]^

**Figure 3:**
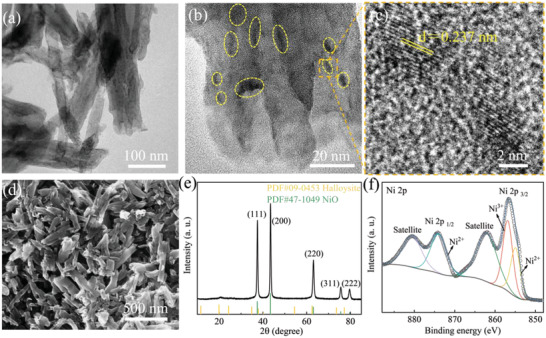
a) TEM, b) HRTEM, c) SEM image, and d) XRD pattern of the e) NiO@Halloysite composite. High‐resolution XPS spectra of f) Ni 2*p* for the NiO@Halloysite composite.

After the *β*‐Ni(OH)_2_@halloysite particles were calcined at 550 °C under argon flow, NiO@Halloysite composite was obtained, and the nanotube structure of halloysite particles is preserved as seen in **Figure** **3**a. Furthermore, the presence of NiO particles (indicated by yellow circles with dash lines) is proved with the TEM micrograph in Figure 3b. The diameters of *β*‐Ni(OH)_2_ and NiO nanoparticles in the synthesized *β*‐Ni(OH)_2_@halloysite and NiO@Halloysite composites were counted and calculated based on 150 nanoparticles in the TEM images. Figure S5 (Supporting Information) shows the particle size distributions of *β*‐Ni(OH)_2_ and NiO nanoparticles in the corresponding composites, indicating *β*‐Ni(OH)_2_ and NiO nanoparticles possess a small particle size centred in the range of 4 to 12 nm. The *d*‐spacing of NiO in NiO@Halloysite composite is calculated to be ≈0.237 nm from its HRTEM image (Figure 3c), which is in good agreement with the (111) plane spacing in NiO crystal phases.^[^ ^37^ ^]^ Figure 3e shows the XRD patterns of the NiO@Halloysite composite, exhibiting strong peaks of 2*θ* at ≈37.46°, 43.50°, 63.11°, 75.55°, and 79.68° which are corresponding to the (111), (200), (220), (311), and (222) atomic planes in NiO (PDF#47‐1049), respectively.

The scanning electron microscope (SEM) image shown in Figure 3d exhibits the morphology of NiO@Halloysite composite. It is observed that the nanorod structure of the halloysite is well‐preserved after being deposited with NiO. Therefore, the modification with NiO on the halloysite nanotubes could increase the number of active reaction sites and form heterostructure on the surface of composites. Moreover, energy dispersive spectroscopy (EDS) elemental mapping analysis verified Ni element's presence and its uniform distribution in the synthesized composite samples, as shown in Figure S6 (Supporting Information). It illustrates the homogeneous distribution of Al, C, Si, and O elements in the sample, which originate from aluminosilicate nanotubes in halloysites. X‐ray photoelectron spectroscopy (XPS) was further applied to analyze the elemental components and chemical binding states of NiO@Halloysite. Figure S7 (Supporting Information) shows the full XPS spectra demonstrating the coexistence of Ni, O, C, Si, and Al in the NiO@Halloysite composite. Figure 3f displays the high‐resolution Ni 2*p* XPS spectra of the NiO@Halloysite composite, which shows Ni 2*p*_1/2_ with two peaks at ≈880 and ≈874 eV, corresponding to satellite and Ni^2+^, respectively. In addition, the main peaks at ≈862, ≈856, and ≈854 eV of Ni 2*p*_3/2_ are related to satellite, Ni^3+^ and Ni^2+^, respectively, proving the existence of NiO in the synthesized composites.^[^ ^38^ ^]^

**Figure 4:**
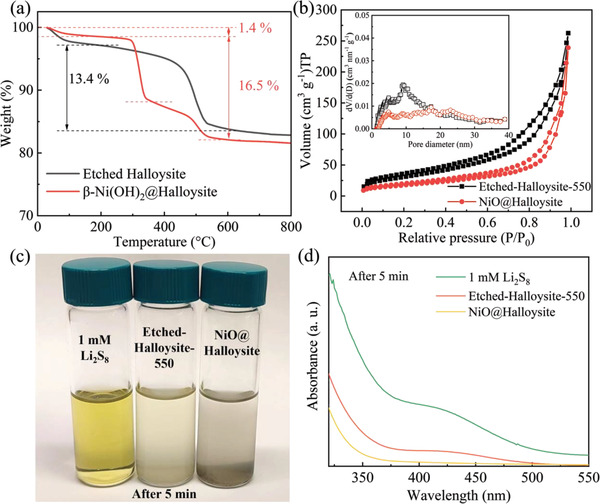
a) TGA curves of the etched halloysite and *β*‐Ni(OH)_2_@halloysite in synthetic air, b) N_2_ adsorption/desorption isotherms of Etched‐Halloysite‐550 and NiO@Halloysite composites with the inset corresponding to the Barrett–Joyner–Halenda (BJH) pore size distribution plot. c) The optical photograph and d) UV–vis spectroscopy of 1 mm Li_2_S_8_ in DOL/DME (1:1 v/v) solution and the solutions after adding Etched‐Halloysite‐550 and NiO@Halloysite for aging 5 min with the same surface area of 1.5 m^2^‐g^−1^ calculated based on their BET‐specific surface area results.

The thermogravimetric analysis (TGA) was conducted to investigate the stoichiometric composition of the synthesized composite and the results are exhibited in **Figure** **4**a, indicating the *β*‐Ni(OH)_2_@halloysite composite shows three main regions of weight loss with increasing temperature. The first region, from room temperature to 180°C, is caused by the loss of physically adsorbed water on the surface of composites. Then, when the temperature rises from 180 to 360°C, the *β*‐Ni(OH)_2_ will convert to NiO.^[^ ^39^ ^]^ The third weight loss region is from 360 to 600°C, it can be explained by the dehydration process of interlayer water in the halloysite particles, which can also be seen in the etched halloysite sample.^[^ ^21^, ^40^ ^]^ According to TGA result, the content of *β*‐Ni(OH)_2_ in the composite is calculated and estimated to be ≈51.8 wt.%. The equation and calculation details are shown in Note S1 (Supporting Information). Figure 4b illustrates the N_2_ adsorption–desorption curves of the calcined halloysite material and NiO@Halloysite composite. The adsorption isotherms of both samples show Type IV isotherm. The sharp knee‐bend and hysteresis loop at higher P/P_0_ are observed in the isotherm, demonstrating the existence of mesopores in Etched‐Halloysite‐550 and NiO@Halloysite composite because the hysteresis loop is related to the capillary condensation and evaporation occurring at mesopores.^[^ ^41^ ^]^ Both samples show a relatively wide pore size distribution in the range of 5–40 nm, confirming mesopores' presence in these materials. It is worth noting that these mesopores would be beneficial for sulfur loading and polysulfide confinements.^[^ ^42^ ^]^ Compared with the Etched‐Halloysite‐550, the pores size distribution range of NiO@Halloysite composite does not change, while the number of pores in the range from 5 to 15 nm decreases, indicating NiO particles enter into the halloysite nanotubes. The specific surface areas of calcinated halloysite and NiO@Halloysite composite samples were determined by the Brunauer–Emmett–Teller (BET) method. The BET equation is shown in the Experimental Section, and the calculation results of the specific surface area are shown in Table S1 (Supporting Information). The calcinated halloysite and NiO@Halloysite show a specific surface area of 114.3 and 64.7 m^2^ g^−1^, respectively. The decrease in specific surface area can be ascribed to the occupation of NiO particles in halloysite pores.

The static adsorption test investigated the adsorption capability of Etched‐Halloysite‐550 and NiO@Halloysite to polysulfides. Based on the BET‐specific surface area test results, different amounts of Etched‐Halloysite‐550 and NiO@Halloysite particles with the same surface area (1.5 m^2^ g^−1^) were added into 4 mL Li_2_S_8_ solution (1 mM) and aged in the glovebox. Figure 4c shows the optical photo of the adsorption results after aging for 5 min. It can be observed that the solution added with NiO@Halloysite immediately changed from yellow to colorless after aging 5 min, while the solution added with Etched‐Halloysite‐550 was still light yellow, suggesting the adsorption process was more efficient and faster in the presence of additional NiO particles. Moreover, the ultraviolet–visible (UV–vis) spectra (Figure 4d) of corresponding supernatants shows that the absorbance peak of Li_2_S_8_ at ≈400–450 nm disappeared in the solution containing NiO@Halloysite after 5 min's adsorption, while remained in the solution adding with Etched‐Halloysite‐550. It further confirmed the better LiPSs affinity of NiO@Halloysite. After aging for 1 h in the glovebox, as shown in Figure S8 (Supporting Information), both solutions change to colorless, and the absorbance peak of Li_2_S_8_ disappeared, indicating that the LiPSs in the solution has been fully adsorbed.

To investigate the electrochemical performance of halloysite and NiO@Halloysite, sulfur was loaded on these host materials and subsequently used as cathodes in Li–S batteries. The sulfur content in the Etched‐Halloysite‐550 and NiO@Halloysite composites are 73.5 and 70.3 wt.%, respectively, determined from TGA curves in Figure S9 (Supporting Information). The electrochemical impedance spectroscopy (EIS) measurements were conducted after assembling the Li–S half‐cells (see section Electrochemical Measurements) and stabilizing for 12 h; after that, tested with a sinusoidal alternating voltage perturbation of 5 mV, and the alternating current response of the system was recorded within the frequency range from 10^6^ to 0.01 Hz at open circuit voltage. EIS was used to separate and quantify the simultaneously occurring processes on a complex heterogenous interface that would otherwise be indistinguishable from one another with other methods, such as CV or GCD.^[^ ^43^ ^]^ It should be noted that EIS spectra are measured for the Li–S half‐cells, meaning that all components of the test cells, i.e., proposed cathodes, Li foil anode, Celgard 2700 separator, will contribute to the complex plane plots characteristics. Furthermore, it is essential to note that the EIS results measured in the two‐electrode system should under no circumstances be interpreted as the sole characterization of cathode material. However, we can observe changes in the system in general, which is a related structural‐property relationship.

**Figure 5:**
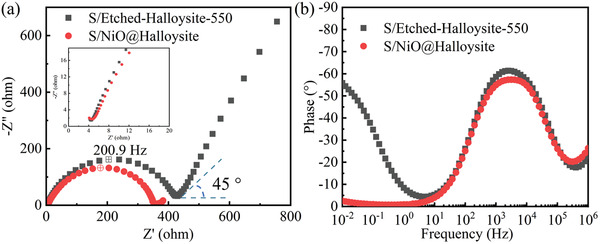
EIS data was recorded for Li–S half‐cells based on S/Etched‐Halloysite‐550 and S/NiO@Halloysite cathodes. a) Complex plane plot and inset for enlarged spectra at the high‐frequency range, b) phase angle versus frequency plots.

**Figure** **5** displays the complex plane and phase angle plots conducted for two electrodes Li–S half‐cell systems based on S/Etched‐Halloysite‐550 and S/NiO@Halloysite cathodes at open circuit voltage, demonstrating great electrochemical wetting has been established for both systems. Graphical analysis of complex plane plots shows depressed semicircles at high and middle frequencies (from 10^6^ to 10 Hz), which can be ascribed to coupling double‐layer capacitance and the faradaic process at high‐frequencies. The Li–S coin cell based on the S/NiO@Halloysite cathode has a smaller semicircle diameter than that for the S/Etched‐Halloysite‐550 cathode, indicating that the S/NiO@Halloysite‐based system has lower charge‐transfer resistance and faster charge transfer process at the heterogeneous surface (0° phase shift between potential and current signals). The quicker charge transfer kinetic properties of the S/NiO@Halloysite cathode could attribute to the good adsorption ability and affinity toward LiPSs of NiO.^[^ ^29^ ^]^ The characteristic frequency of these two systems is shown in Figure 5a, both at 200.9 Hz, indicating similar electron transfer processes in Li–S coin cells based on the S/Etched‐Halloysite‐550 and S/NiO@Halloysite cathodes.^[^ ^44^ ^]^ The linear region in the complex plane at low frequencies (from 10 to 0.01 Hz) for the S/Etched‐Halloysite‐550 cathode is related to the deviation from solely Warburg‐like impedance behavior due to Li^+^ ion diffusion into the mesoporous S/Etched‐Halloysite‐550 cathode structure. The phase angle plot in Figure 5b shows a well‐defined broad peak corresponding to the semicircle at frequencies >10 Hz and a line going asymptotically to −55° and 0° for the S/Etched‐Halloysite‐550 and S/NiO@Halloysite at frequencies < 10 Hz, respectively. This finding confirms that the mechanism changes from a diffusion‐limited process to a charge transfer‐limited process due to surface morphology (observed by N_2_ physisorption, SEM, TEM) and additional reaction sites (observed by TEM, XPS). In other words, the surface morphology of the S/Etched‐Halloysite‐550 electrode is geometrically rougher (e.g., pores, defects, etc.) and after the modification with NiO on the halloysite, the surface is somewhat smoother and the reaction site properties (e.g., rate of charge transfer) are heterogeneously distributed. The inset in Figure 5a represents the enlarged complex plane plot shifted to higher values by constant resistance ≈4.2 Ω for both systems. The phase angle versus frequency plot shown in Figure 5b indicates that the S/NiO@Halloysite cathode exhibits lower phase angle at low frequencies than that of the S/Etched‐Halloysite‐550 cathode, suggesting higher ionic permeability of the S/NiO@Halloysite cathode.^[^ ^44^ ^]^

**Figure 6:**
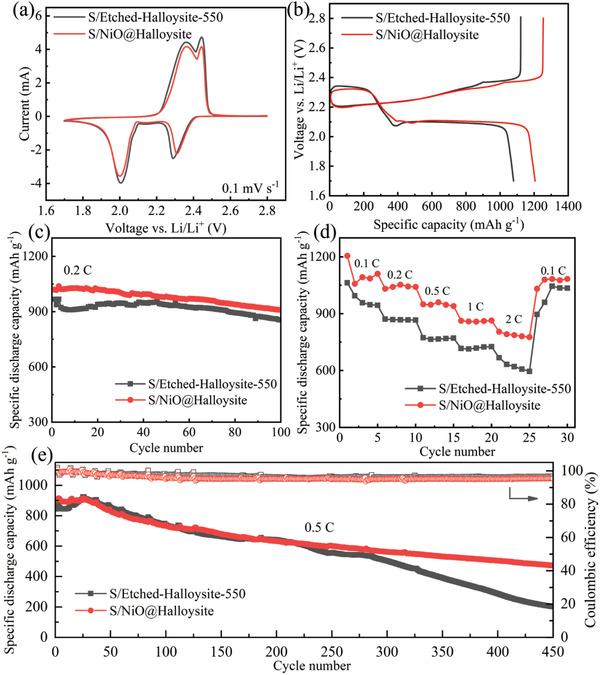
a) The third cycle of CV curves scanned at 0.1 mV s^−1^. b) Galvanostatic charge–discharge curves at 0.1 C, c) cycling stability at 0.2 C for 100 cycles, d) rate capability, and e) long‐term cycling stability of Li–S batteries at 0.5 C for 450 cycles based on the S/Ethched‐Halloysite‐550 and S/NiO@Halloysite cathodes in the electrolyte containing 1 m LiTFSI and 2 wt.% of LiNO_3_ in DME/DOL (1:1 v/v) solution.

The CV and GCD tests were performed to demonstrate the electrochemical performance of the S/Etched‐Halloysite‐550 and S/NiO@Halloysite cathode in Li–S cell. **Figure** **6**a shows the characteristic CV shape of these cathodes in the third cycle within the voltage range of 1.7–2.8 V versus Li/Li^+^ at a potential scan rate of 0.1 mV s^−1^. The anodic and cathodic scans showed a pair of well‐defined redox peaks, demonstrating that sulfur's electrochemical reduction and oxidation occur in two stages. The anodic peaks at ≈2.30 and 2.01 V are associated with the reduction of elemental sulfur to LiPSs (Li_2_S_n_, 4≤*n*<8) and to short‐chain Li_2_S/Li_2_S_2_.^[^ ^45^ ^]^ The cathodic peaks at ≈2.37 and 2.44 V are related to reversible oxidation reactions to form Li_2_S/Li_2_S_2_ to Li_2_S*_n_* and finally to S_8_.^[^ ^22^ ^]^ It is noted that the S/NiO@Halloysite cathode exhibits a higher reduction peak (≈2.31 V) than S/Ethched‐Halloysite‐550 (≈2.29 V), indicating better transformation kinetics and electrocatalyst performance by depositing NiO on the surface of halloysite. Moreover, the first three cycles of CV curves shown in Figure S10 (Supporting Information) are almost overlapped, indicating good cycling stability of the S/NiO@Halloysite cathode. The CV and EIS results confirm the better electrochemical performance of the S/NiO@Halloysite cathode and suggest that the electroactivity and affinity to LiPSs of NiO rather than the specific surface area plays an important role in the electrochemical reaction process. The GCD curves of the S/Etched‐Halloysite‐550 and S/NiO@Halloysite at 0.1 C are displayed in Figure 6b. It reveals that the initial specific discharging capacity of the S/NiO@Halloysite cathode is much higher than that for the S/Etched‐Halloysite‐550 cathode, which is 1205.47 and 1079.25 mAh g^−1^, respectively.

Figure 6c presents the cycling stability of both cathodes at 0.2 C for 100 cycles, the specific discharging capacity of the S/Etched‐Halloysite‐550 cathode decreases from 967.24 mAh g^−1^ to 855.13 mAh g^−1^. In contrast, the S/NiO@Halloysite cathode still delivers 908.91 mAh g^−1^ after 100 cycles, revealing better cycling stability. The rate capabilities were further investigated at 0.1, 0.2, 0.5, 1, and 2 C as shown in Figure 6d. The S/NiO@Halloysite cathode delivers the discharging capacity of 1205.47, 1031.85, 949.52, 862.66, and 791.88 mAh g^−1^ at 0.1, 0.2, 0.5, 1, and 2 C, respectively, which are higher than that of the S/Etched‐Halloysite‐550 cathode. When the current density changed to 0.1 C, the S/NiO@Halloysite cathode retains a specific discharge capacity of 1079.87 mAh g^−1^.

The long‐term cycling performance of the coin cell with S/NiO@Halloysite and S/Etched‐Halloysite‐550 cathodes was measured at 0.5 C for 450 cycles, as shown in Figure 6e. The S/NiO@Halloysite cathode still preserves a discharging capacity of 467.45 mAh g^−1^ after 450 cycles, while the discharging capacity of S/Etched‐Halloysite‐550 rapidly decreases to 192.80 mAh g^−1^ after 450 cycles. Moreover, the Coulombic efficiency of the S/NiO@Halloysite cathode maintains >95% during the long‐term cycling process, indicating good cycling performance and electrochemical reversibility. The superior long‐term cycling performance of the S/NiO@Halloysite cathode is presumably due to the combination of physical confinements of tubular halloysites and chemical confinements of NiO. Therefore, the proposed NiO@Halloysite provides a good example of the application of halloysite in electrochemical energy storage electrodes and can be extended to other abundant nanoclay materials.

## 3 Conclusion

In summary, a strategy of using natural clay mineral halloysite as a sulfur host material and uniform deposition of NiO nanoparticles into halloysite nanotubes to promote their LiPSs affinity and electrochemical performance is proposed. The Li–S batteries based on the S/NiO@Halloysite composite cathode delivered a specific discharge capacity of 1205.47 mAh g^−1^ at 0.1 C, which is higher than that of the S/Etched‐Halloysite‐550 cathode. Moreover, the S/NiO@Halloysite also showed better rate capability and long‐term cycling performance that a specific discharge capacity of 467.45 mAh g^−1^ remained after charging and discharging at 0.5 C for 450 cycles. The improvements in electrochemical performance of Li–S battery with S/NiO@Halloysite cathode can be attributed to the strong adsorption ability of NiO and the great physical confining and additional adsorption ability of halloysite nanotube, thereby effectively inhibiting the dissolution and migration of lithium polysulfides. It demonstrates that halloysite and other natural clay minerals can provide a cost‐effective component for sulfur host materials in Li–S batteries and exhibits application prospects in other energy storage systems.

## 4 Experimental Section

### Materials and Reagents

Esan Eczacıbaşı Industrial Raw Materials Company supplied halloysite. Sulfuric acid (H_2_SO_4_, 98%), nickel chloride hexahydrate (NiCl_2_·6H_2_O, 99.9%), ammonium chloride (NH_4_Cl, 99.5%), ammonia solution (NH_3_·H_2_O, 28 wt.%), anhydrous ethanol, lithium nitrate (LiNO_3_, 99.99%), bis(trifluoromethane) sulfonimide lithium salt (LiTFSI, anhydrous, 99.99%), *N*‐methyl‐2‐pyrrolidone (NMP, anhydrous, 99.5%), polyvinylidene fluoride (PVdF), 1, 2‐dimethoxyethane (DME, anhydrous, 99.5%), sulfur powder, and 1, 3‐dioxolane(DOL, anhydrous, 99.8%) were purchased from Sigma–Aldrich. All reagents and materials were utilized without any further treatments or purifications.

### Acid Pretreatment of Halloysite Nanotubes

The halloysite (1 g) was dispersed in sulfuric acid solution (100 mL, 1 mol L^−1^) and heated at 50 °C for 18 h. Then, the mixture was washed and centrifuged with distilled water several times until the supernatant reaches neutral.

### Synthesis of NiO@Halloysite Composite

Halloysite solution (10 mL) (1 mg mL^−1^) was prepared by dispersing etched halloysite nanotubes in water and ultrasonicated for 30 min. NiCl_2_·6H_2_O (0.5 mmol) (0.0648 g) and 5 mmol NH_4_Cl (0.267 g) were added into the halloysite solution, and the mixed solution was dispersed by ultrasonication for 30 min. Then, 0.5 mL ammonia solution was dropwise added and stirred for 10 min followed by transferred into an autoclave. After the hydrothermal treatment at 140°C for 12 h, light‐green products were collected and washed by centrifugation with ethanol for three times and dried at 60°C. The products were calcined in the Muffle furnace at 550°C for 1 h with a heating rate of 5°C min^−1^ to get NiO@Halloysite composite. As a reference, the acid‐pretreated halloysite was also calcinated at 550°C for 1 h, which was assigned as Etched‐Halloysite‐550.

### Preparation of Sulfur Composite Cathode

The sulfur powder was mixed with NiO@Halloysite composite (the mass ratio is 7:3) by grinding for 30 min. Then, a Teflon container was used to seal the mixed powder and heated in a tube furnace at 155 °C for 12 h under an argon atmosphere to load S into the Etched‐Halloysite‐550 and NiO@Halloysite.

### Characterization

The SEM (LEO Gemini 1530 microscopy) equipped with an energy dispersive spectroscopy (EDS, Thermo Fisher) was operated at 7 kV to observe the morphology of materials. Before the measurement, all samples were sputtered with a layer of amorphous carbon (≈5 nm). TEM (JEOL JEM‐2100 instrument) measurements were conducted at 200 kV. XRD (Bruker D8, with Cu K_*α*_ radiation) was performed to investigate the crystal structures of samples. PerkinElmer (TGA 8000) was utilized to analyze the thermogravimetric curves of samples, which were conducted in 30–800 °C under synthetic air with a heating rate of 10°C min^−1^. Lambda 650 spectromete (PerkinElmer) was used to measure the UV–vis spectra (300–800 nm). XPS (ESCA‐Lab‐220i‐XL, Thermo Fisher Scientific) measurement was conducted with Al K_*α*_ sources (*hν* = 1486.6 eV). Nitrogen (N_2_) adsorption–desorption measurements (Quantachrome Autosorb‐1 systems) were performed at a temperature of 77 K, and the BET method with multipoint analysis was utilized to calculate the samples’ specific surface areas, in which the BET equation was:^[^ ^46^ ^]^

(1) $$\begin{array}{c} \frac{1}{W[P/P_{0}-1]}=\frac{1}{W_{m}C}+\frac{C-1}{W_{m}C}\left(\frac{P}{P_{0}}\right) \end{array}$$

where *P* is the adsorbate equilibrium pressure, *C* is a constant, and *W* and *W*_m_ are the weight of adsorbed adsorbate and the monolayer adsorbed adsorbate, respectively. According to Equation 1, the values of *W*_m_ could be calculated, and the specific surface area of the sample is calculated based on Equation 2:^[^ ^47^ ^]^

(2) $$\begin{array}{c} S_{\mathrm{BET}}=\frac{W_{m}\cdot N_{A}\cdot A_{m}}{V_{0}\cdot m} \end{array}$$

where *N*_A_ is the Avogadro constant, *A*_m_ is the molecular cross‐sectional area of adsorbate, *V*_0_ is the molar gas volume of adsorbate at STP (standard temperature and pressure), and *m* is the mass of sample.

### Lithium Polysulfides Adsorption Tests

To prepare Li_2_S_8_ solution for the adsorption test, S and Li_2_S power with a molar ratio of 5:1 were dissolved in the mixed solution, DME and DOL with the ratio of 1:1 v/v, and stirred for 48 h at 80°C in the glove box. Then, taking out 4 mL Li_2_S_8_ solution with a concentration of 1 mM in two sample bottles and adding different amounts of Etched‐Halloysite‐550 and NiO@Halloysite with the same surface area (1.5 m^2^ g^−1^, calculated from BET‐specific surface area results in Table S1), respectively. Taking pictures and observing the changes in different samples after aging for 5 min and 1 h in the glove box, the UV–vis spectroscopy (Lambda 650 spectrometer, PerkinElmer) test was performed to the supernatant solutions.

### Electrochemical Measurements

The electrochemical performance tests (CV, GCD, and EIS) of Li–S batteries were conducted by CR2032 coin cells. To prepare the cathode of the coin cells, the conductive carbon black, PVdF and S/NiO@Halloysite composites or S/Ethced‐halloysite‐550 were mixed by grinding in the mortar with a ratio of 7:2:1 (in mass) in NMP solution. Carbon paper was used as current collector and coating with the cathode slurry by the doctor blade method, the coated electrodes were dried at 60°C in a vacuum oven for 12 h. Then, the Ar‐filled glovebox (UNIlab plus, M. BRAUN) was utilized to assemble the coin cells, which using Li foil as an anode, the prepared cathodes, Celgard 2700 a separator and 40 µL electrolyte. For preparing the electrolyte, 1 m LiTFSI was dissolved in DME/DOL solution (1:1 v/v) and adding 2 wt.% of LiNO_3_. Before performing the electrochemical measurements, the assembled coin cells were aged for 12 h at room temperature and with open circuit potential to allow a better and more stable electrode–electrolyte interface. GCD was conducted with the Neware battery testing system (CT‐4008‐5 V10 mA) between 1.7–2.8 V versus Li/Li^+^ at 25.0°C. CV was conducted by a Biologic VMP3 electrochemical workstation. EIS was performed using GAMRY Interface 1000 by applying a voltage perturbation of 5 mV between 10^6^ and 0.01 Hz, assembled coin cells were aged for 12 h and tested at open circuit potential. The specific discharging capacity of different samples was calculated according to the sulfur's mass in the cathode, and the areal mass loading of sulfur in fabricated cathodes was ≈2 mg cm^−2^, and the current density of 1 C equals 1675 mA g^−1^.

## Conflict of Interest

The authors declare no conflict of interest.

## Supporting information

Supporting Information

## Data Availability

The data that support the findings of this study are available in the supplementary material of this article.
