# Supplementary material for: Nickel Oxide Decorated Halloysite Nanotubes as Sulfur Host Materials for Lithium–Sulfur Batteries
Source: Glob Chall. 2023 May 13;7(7):2300005. doi: 10.1002/gch2.202300005 (PMC10362100; doi:10.1002/gch2.202300005)
Supplement: Supplementary file 1 — Supporting Information [file GCH2-7-2300005-s001.pdf]

Global Challenges
Open Access

Supporting Information

for *Global Challenges.*, DOI 10.1002/gch2.202300005

Nickel Oxide Decorated Halloysite Nanotubes as Sulfur Host Materials for Lithium–Sulfur Batteries

*Meltem Karaismailoglu Elibol, Lihong Jiang, Dongjiu Xie, Sijia Cao, Xuefeng Pan, Eneli Härk and Yan Lu**

Supporting Information

Nickel oxide decorated halloysite nanotubes as sulfur host materials for lithium-sulfur batteries

*Meltem Karaismailoglu Elibol, Lihong Jiang, Dongjiu Xie, Sijia Cao, Xuefeng Pan, Eneli Härk, Yan Lu**

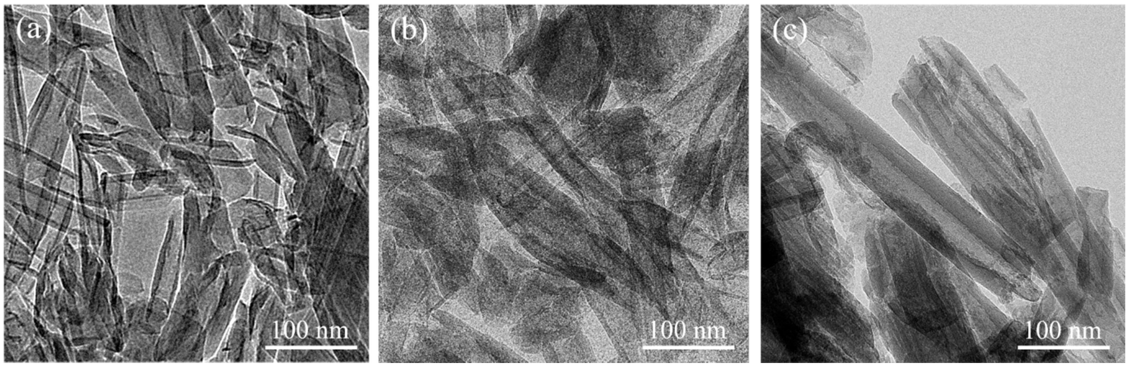

Figure S1. TEM images of the raw halloysite (a), etched halloysite (b), and etched-halloysite after calcination at 550 °C (c).

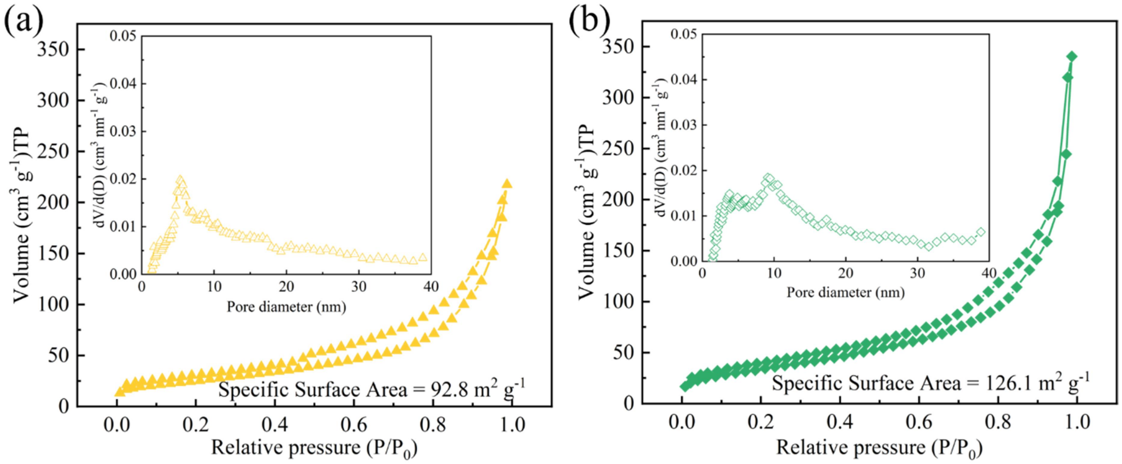

Figure S2. N_2 adsorption/desorption curves of (a) the raw halloysite and (b) etched halloysite with the inset corresponding to the pore size distribution plot.

Table S1 The BET-specific surface area results of samples

	Specific surface area ($m^2\ g^{-1}$)
Raw halloysite	92.8 ± 3.2
Etched halloysite	126.1 ± 9.5
Ethched-Halloysite-550	114.3 ± 4.1
NiO@Halloysite	64.7 ± 4.7

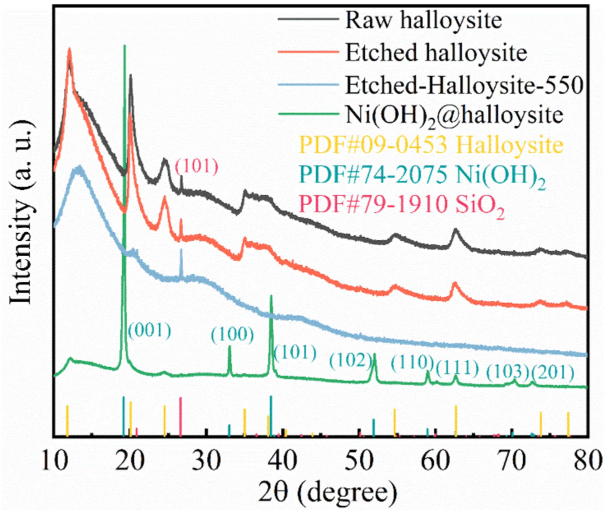

Figure S3. XRD spectra of the raw, etched halloysite, etched halloysite after calcination at 550 °C and the β-$Ni(OH)_2$@halloysite composite.

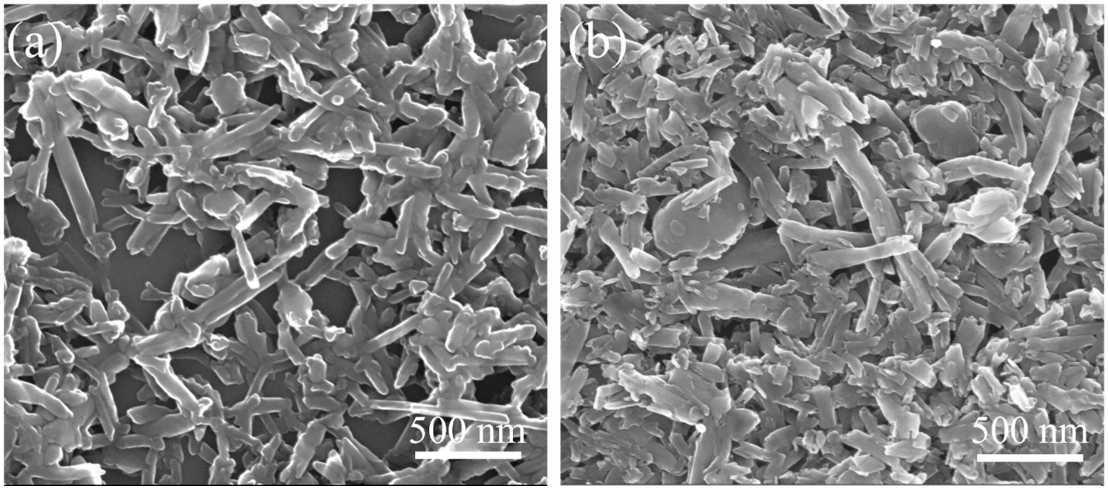

Figure S4. SEM images of the etched halloysite (a) and β-$Ni(OH)_2$@halloysite composites (b).

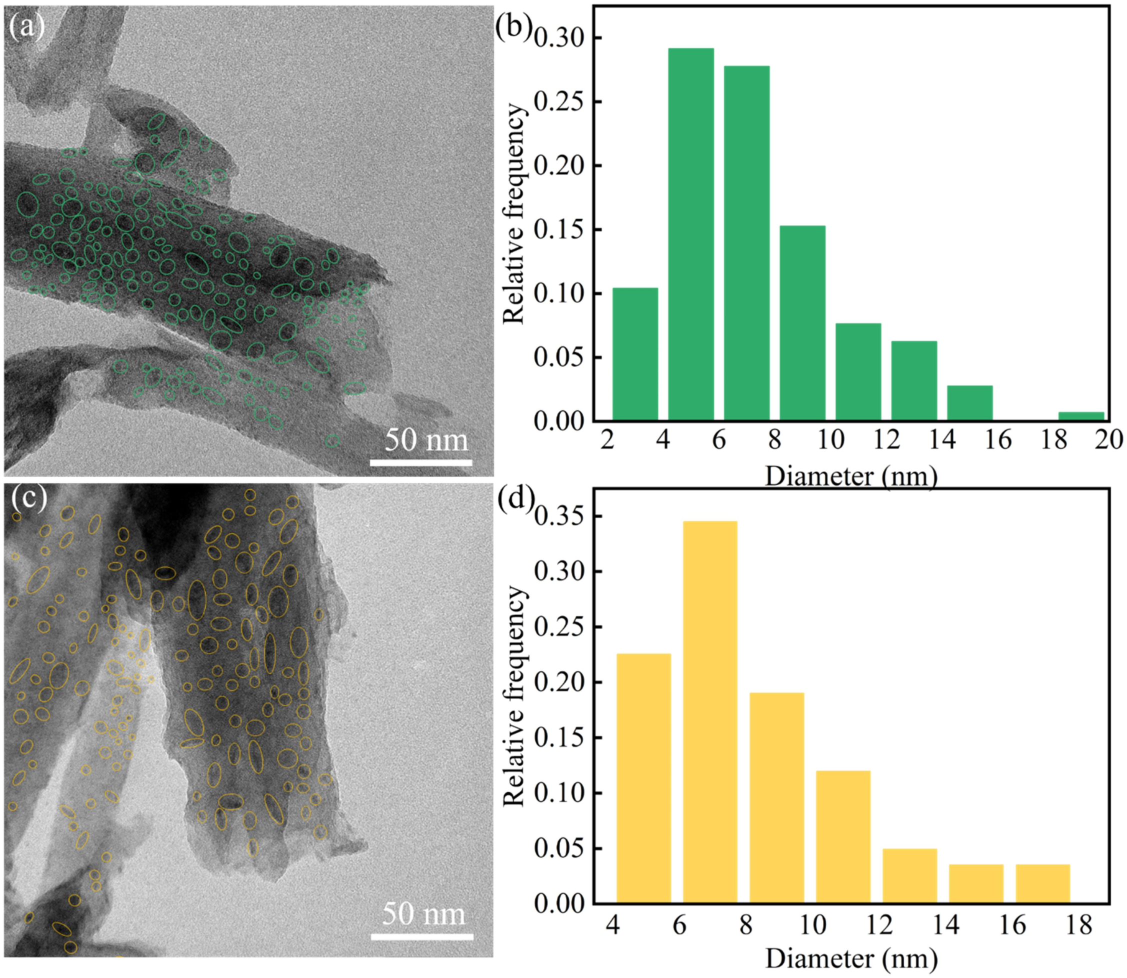

Figure S5. TEM images of the β-$Ni(OH)_2$@halloysite (a) and NiO@Halloysite composites (c), and particle size distributions of the β-Ni(OH)2 (b) and NiO nanoparticles (d) in the corresponding composites.

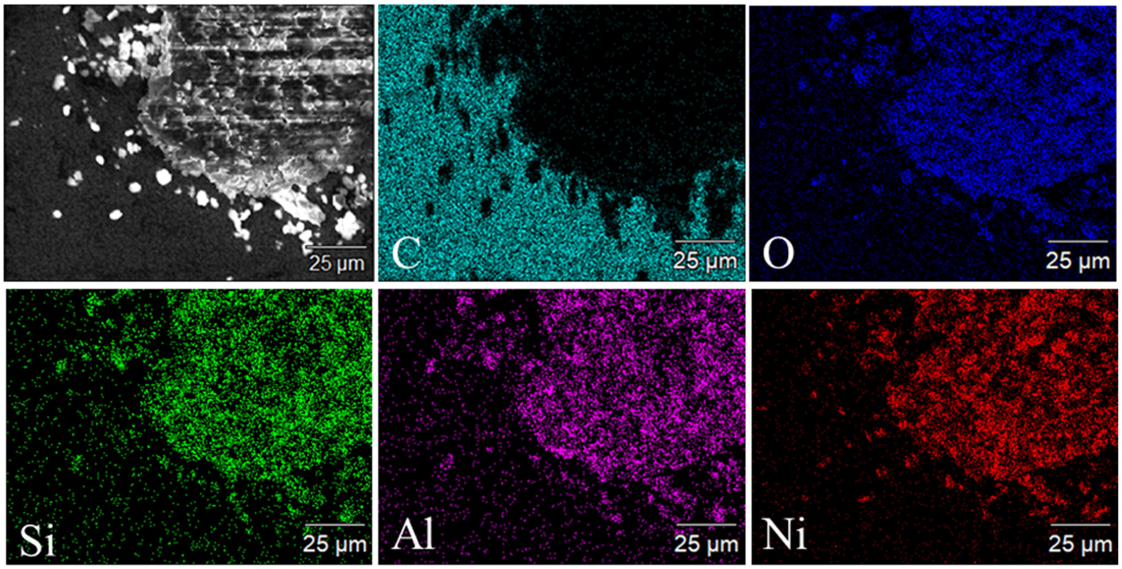

Figure S6. SEM-EDS elemental mapping of the NiO@Halloysite composite.

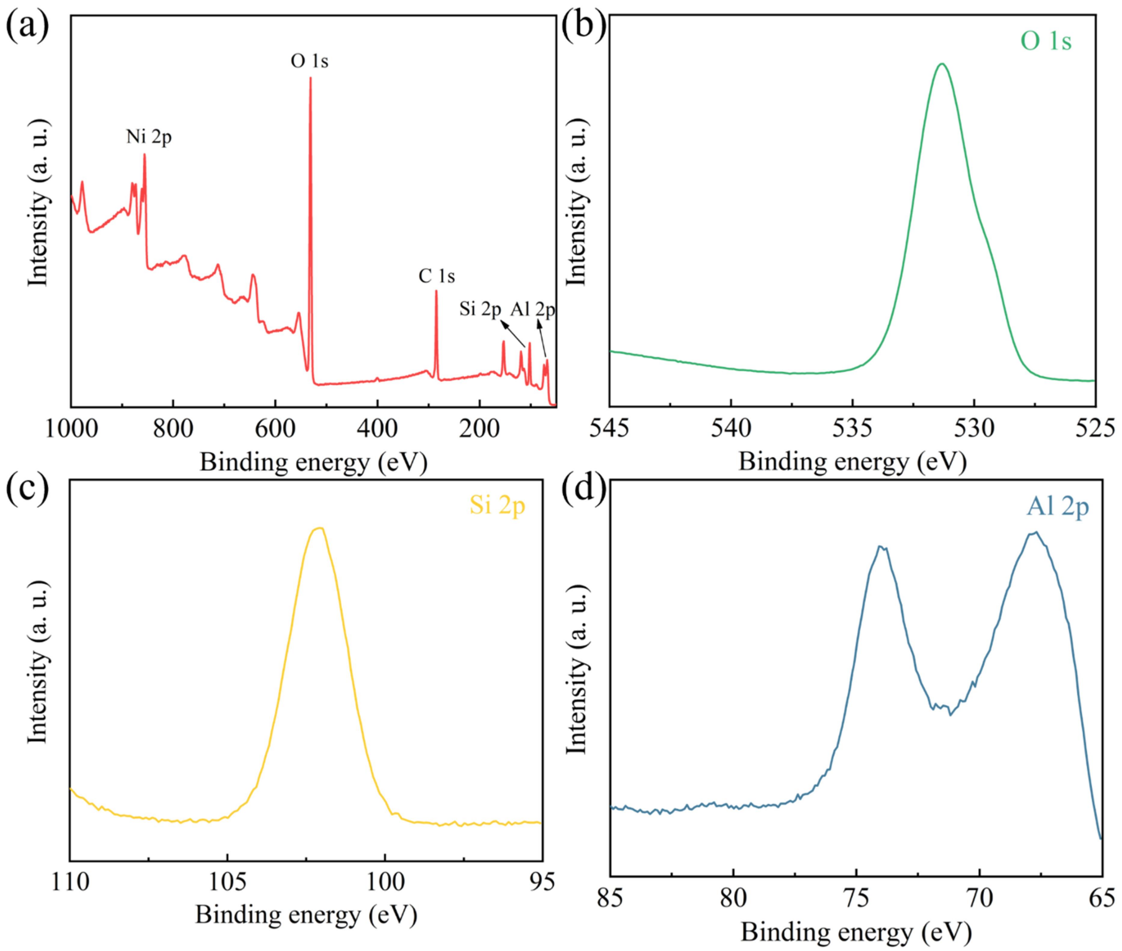

Figure S7. Full XPS spectra (a), high resolution of O 1s (b), Si 2p (c), and Al 2p (d) spectra of the NiO@Halloysite composite.

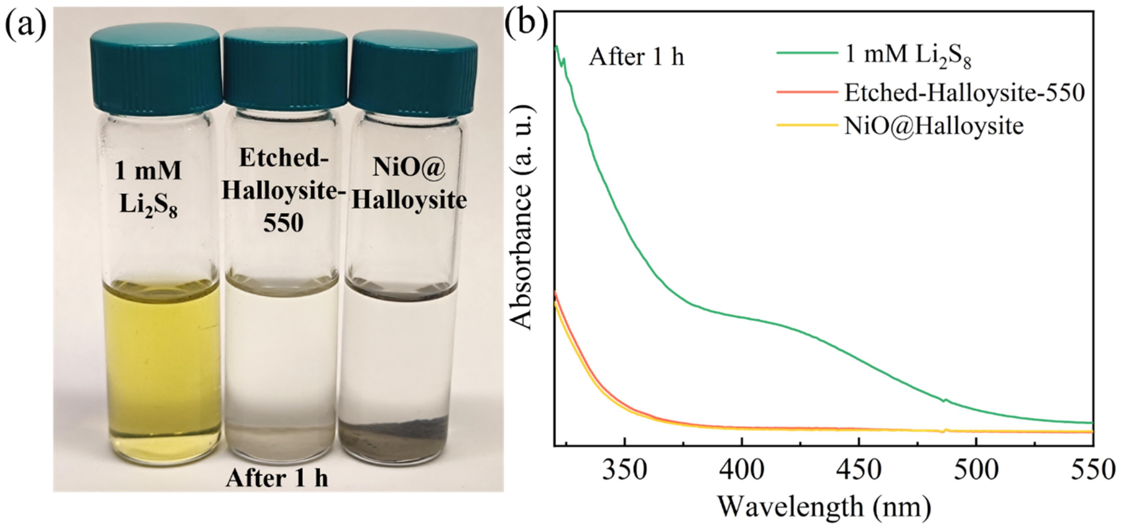

Figure S8. The optical photograph (a) and UV-vis spectroscopy (b) of 1 mM Li_2S_8 in DOL/DME (1:1 v/v) solution and the solutions after adding Etched-Halloysite-550 and NiO@Halloysite for aging 1 hour.

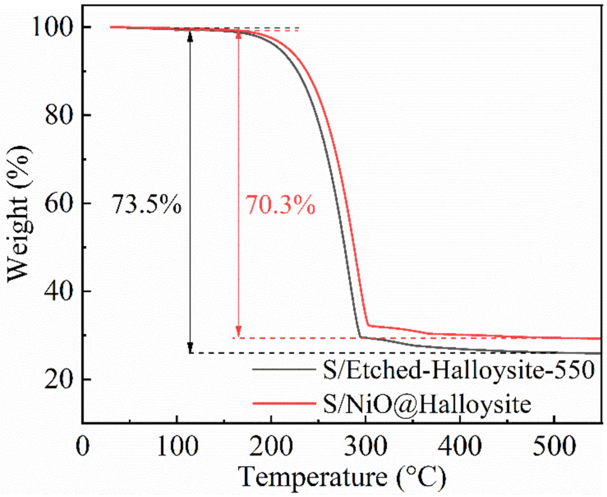

Figure S9. TGA curves of the S/Etched-Halloysite-550 and S/NiO@Halloysite composite.

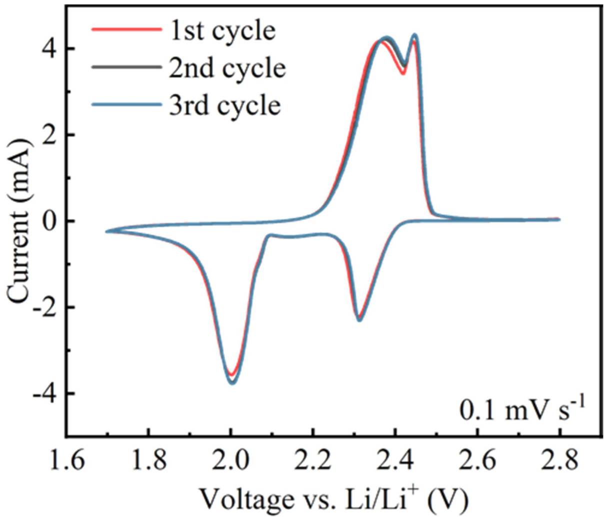

Figure S10. CV curves of the S/NiO@Halloysite composite cathode scanned at 0.1 mV s^{-1}.

Note 1. The calculation for the stoichiometric composition of β-$Ni(OH)_2$ in the composite

The weight loss equation for the β-$Ni(OH)_2$@halloysite composite could be:

$$(1-a)\cdot m_{\mathrm{Hal}} + a\cdot m_{\mathrm{Ni(OH)_2}} = m_{\mathrm{Com}}$$

Where a is the mass percentage of β-$Ni(OH)_2$ in the β-$Ni(OH)_2$@halloysite composite. m_{Hal} is the normalized weight loss percentage of interlayer water in etched-halloysite (13.8 wt%). $m_{\mathrm{Ni(OH)_2}}$ is the theoretical weight loss percentage for the transition from $Ni(OH)_2$ to NiO (19.4 wt%). m_{Com} is the normalized weight loss percentage of β-$Ni(OH)_2$@halloysite composite (16.7 wt%).
